# Preliminary phytochemical and elemental analysis of aqueous and fractionated pod extracts of *Acacia nilotica *(Thorn mimosa)

**Published:** 2014

**Authors:** Mohammed Shaibu Auwal, Sanni Saka, Ismail Alhaji Mairiga, Kyari Abba Sanda, Abdullahi Shuaibu, Amina Ibrahim

**Affiliations:** 1 *Department of Veterinary Physiology, Pharmacology and Biochemistry, University of Maiduguri, Maiduguri,Nigeria;*; 2 *Department of Veterinary Physiology and Pharmacology, University of Abuja, Gwagwalada, Nigeria; *; 3 *Department of Veterinary Medicine, University of Maiduguri, Maiduguri, Nigeria; *; 4 *Department of Human Anatomy, College of Medical Sciences, University of Maiduguri, Maiduguri, Nigeria.*

**Keywords:** Atomic absorption, Flame emission, Spectrometry

## Abstract

*Acacia nilotica* (Thorn mimosa) is used locally for various medicinal purposes by traditionalists and herbalists in northeastern Nigeria. Plants products have been used since ancient times in the management of various conditions. The bark of *A. nilotica* has been reported to be used traditionally to manage diabetes, dysentery, leprosy, ulcers, cancers, tumor of the eye, ear and testicles, induration of liver and spleen and also in treatment of various condylomas. The objective of this study is to determine the phytochemical and elemental constituents of the extracts of *A.* *nilotica* pods. Flame emission and atomic absorption spectrometry were also used to determine the presence or absence of micro- and macro-elements in the extracts. Phytochemical analysis of the aqueous, ethyl acetate and N-butanol fractionated portions of the pod extracts of *A. nilotica *revealed the presence of tannins, saponins, flavonoids, carbohydrate, whereas carbohydrates and tannins were the only constituent in the residue portion. Anthraquinones, alkaloids, terpene and steroids were not present in the extracts. The elemental screening revealed the presence of iron, potassium, manganese, zinc, calcium, phosphorous, magnesium, sodium, cadmium and copper. Lead, arsenic and molybdenum were not detected in the pod.

## Introduction

Plants are important source of many drugs including antispasmodics, emetics, anti-cancer, antimicrobials etc. Medicinal plants are claimed to possess antibiotic properties and are also used extensively by the tribal people world-wide. Plants have been known to alleviate various diseases in traditional medicine.^1^

Extraction and phytochemical analysis is the separation of medicinally active portions of plant tissues using universal solvent such as water and selective solvents as alcohols through standard procedures.^2^

*Acacia nilotica* is a tree of 5 to 20 meter high with dense spherical crowns, stem and branchlets usually with dark to black coloured, fissured bark, greyish- pinkish slash, exuding a reddish low quality gum. There are roughly 1,300 species of Acacia worldwide which about 950 species are native to Australia and the remainder spread around the dry tropical to warm temperate regions of both hemispheres, including Africa, southern Asia, and the Americas.^3^

*Acacia nilotica* contain some psychoactive alkaloids of which dimethyltryptamine and N-methyltryptamine are most prominent and useful. Other psychoactive compounds present in the plant include tryptamine, β-carbolines, mesculine, bufoteinine and nicotine. *Acacia nilotica* has been reported to have astringent property and is used by the native Africans in the treatment of such conditions as impotence, tumor of the eye or testicle, dysentery, leprosy, colds, cough, congestion, fever, hemorrhoids, leucorrhoea, opthalmia, sclerosis, smallpox, tuberculosis, and indurations of the liver and spleen.^3^ There are also reports of its usage in the treatment of toothache and typhoid. The objective of this study was to evaluate the phytochemical and elemental constituents of the aqueous and fractionated pod extracts of this plant.

## Materials and Methods

**Plant collection and identification.** Fresh pods of *A. nilotica* were collected in June 2008 from Potiskum, Yobe State, Nigeria. The pods were identified by a taxonomist in Department of Biological Sciences, University of Maiduguri, Maiduguri, Nigeria. The pods were air dried for three weeks under the shade and ground into fine powder. 

**Preparation of aqueous extract. **Three hundred and fifty grams (350 g) of the powdered extract sample were exhaustively extracted with distilled water using reflux method. The crude aqueous extract was concentrated *in vacuo* and a brown colored extract weighing two hundred and sixty three grams (263 g) w/w was obtained. It was thereafter stored in a refrigerator at 4 ˚C until used.^4^

**Fig. 1:**
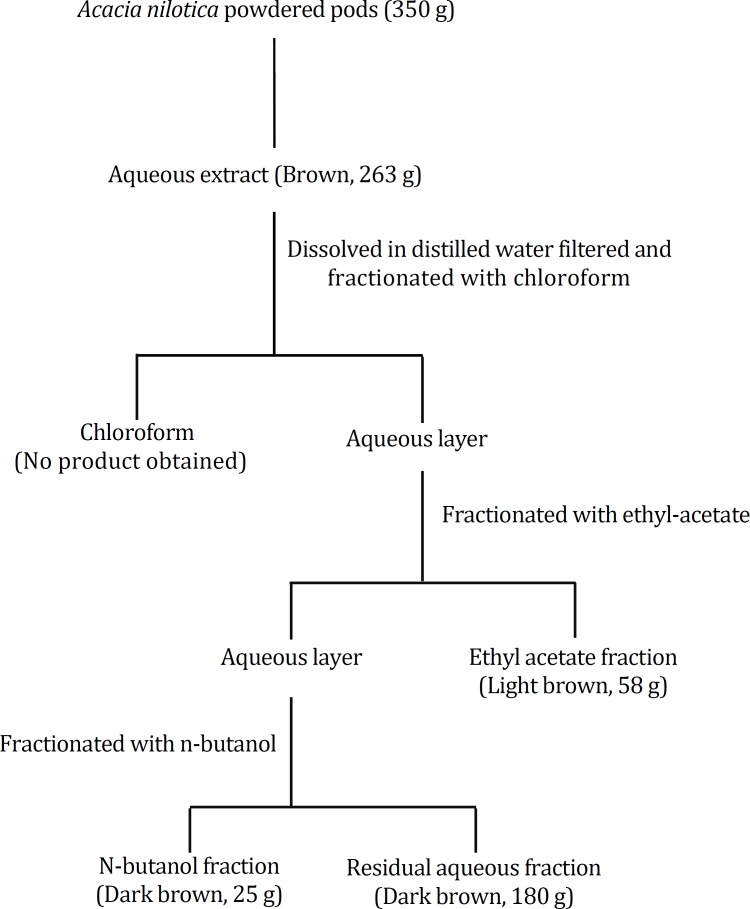
Schematic diagram of extraction and fractionation of *A.* *nilotica* pods extract using various water and various organic solvents

**Fractionation of the aqueous pod extract.** The method used for fractionation of *A. nilotica* pod powder has already been reported.^5^^,^^6^ The crude aqueous pod extract was suspended in cold distilled water and then filtered using Whatman filter paper. The filtrate was thereafter subjected to fractionation using, chloroform, ethyl acetate and n-butanol. The fractionation with the organic solvents of different polarity was done until the organic layers were visibly clear to obtain ethyl acetate (58 g), n-butanol (25 g) soluble fractions and the residue (180 g). The product did not dissolve in chloroform, hence no product was obtained as shown in Fig. 1.

**Phytochemical** **analysis of the** **extracts of *A.*** ***nilotica*.** The aqueous extract and ethyl acetate, N-butanol and residual fractions of *A. nilotica* extracts were subjected to qualitative chemical screening for identification of various classes of active chemical constituents.^7^^, ^^9^

**Test for tannins (Ferric chloride test). **Two millilitres (2 mL) of the aqueous solution of the extract were added to a few drops of 10% Ferric chloride solution (light yellow). The occurrence of blackish blue colour showed the presence of gallic tannins and a green-blackish colour indicated presence of catechol tannins.

**Test for saponins (Frothing Test). **Three millilitres (3 mL) of the aqueous solution of the extract were mixed with 10 mL of distilled water in a test-tube. The test-tube was stoppered and shaken vigorously for about 5 min, it was allowed to stand for 30 min and observed for honeycomb froth, which was indicative of the presence of saponins.

**Test for alkaloids.** One gram (1 g) of the extract was dissolved in 5 mL of 10% ammonia solution and extracted with 15 mL of chloroform. The chloroform portion was evaporated to dryness and the resultant residue dissolved in 15 mL of dilute sulphuric acid. One quarter of the solution was used for the general alkaloid test while the remaining solution was used for specific tests.

**Mayer’s reagent (Bertrand’s reagent). **Drops of Mayer’s reagent was added to a portion of the acidic solution in a test tube and observed for an opalescence or yellowish precipitate indicative of the presence of alkaloids.

**Dragendorff’s reagent. **Two millilitres (2 mL) of acidic solution in the second test-tube were neutralized with 10% ammonia solution. Dragendorff’s reagent was added and turbidity or precipitate was observed as indicative of presence of alkaloids.

**Tests for carbohydrate (Molisch’s test). **A few drops of Molischs solution was added to 2 mL of aqueous solution of the extract, thereafter a small volume of concentrated sulphuric acid was allowed to run down the side of the test tube to form a layer without shaking. The interface was observed for a purple colour as indicative of positive for carbohydrates.

**Tests** **for** **carbohydrate (Barfoed’s** **test). **One milliliter (1 mL) of aqueous solution of the extract and 1ml of Barfoed’s reagent were added into a test-tube, heated in a water bath for about 2 min. Red precipitate showed the presence of monosaccharaides.

**Standard test for combined reducing sugars. **One milliliter (1 mL) of the aqueous solution of the extract was hydrolyzed by boiling with 5 mL of dilute hydrochloric acid (HCl). This was neutralized with sodium hydroxide solution. The Fehling’s test was repeated as indicated above and the tube was observed for brick-red precipitate that indicated the presence of combine reducing sugars.

**Standard** **test** **for** **free** **reducing** **Sugar (Fehling’s** **test).** Two milliliters (2 mL) of the aqueous solution of the extract in a test tube was added into 5 mL mixture of equal volumes of Fehling’s solutions I and II and boiled in a water bath for about 2 min. The brick-red precipitate was indicative of the presence of reducing sugars.

**Test for ketones. **Two millilitres (2 mL) of aqueous solution of the extract were added to a few crystals of resorcinol and an equal volume of concentrated HCl, and then heated over a spirit lamp flame and observed for a rose colouration that showed the presence of ketones.

**Test** **for** **pentoses. **Two millilitres (2 mL) of the aqueous solution of the extract were added into an equal volume of concentrated HCl containing little phloroglucinol. This is heated over a spirit lamp flame and observed for red colouration as indicative of the presence of pentoses.

**Test for phlobatannins (** **HCl** **test). **Two millilitres (2 mL) of the aqueous solution of the extract were added into dilute HCl and observed for red precipitate that was indicative the presence of phlobatannins.

**Test for cardiac glycosides. **Two millilitres (2 mL) of the aqueous solution of the extract was added into 3 drops of strong solution of lead acetate. This was mixed thoroughly and filtered. The filtrate was shaken with 5 mL of chloroform in a separating funnel. The chloroform layer was evaporated to dryness in a small evaporating dish. The residue was dissolved in a glacial acetic acid containing a trace of ferric chloride; this was transferred to the surface of 2 mL concentrated sulphuric acid in a test tube. The upper layer and interface of the two layers were observed for bluish-green and reddish-brown colouration respectively as indicative of the presence of cardiac glycosides.

**Test for steroids (Liebermann-Burchard’s test).** The amount of 0.5 g of the extract was dissolved in 10 mL anhydrous chloroform and filtered. The solution was divided into two equal portions for the following tests. The first portion of the solution above was mixed with one ml of acetic anhydride followed by the addition of 1 mL of concentrated sulphuric acid down the side of the test tube to form a layer underneath. The test tube was observed for green colouration as indicative of steroids.

**Test for steroids (Salkowski’s test). **The second portion of solution above was mixed with concentrated sulphuric acid carefully so that the acid formed a lower layer and the interface was observed for a reddish-brown colour indicative of steroid ring.

**Test for flavonoids (Shibita’s reaction test). **One gram (1 g) of the water extract was dissolved in methanol (50%, 1-2 mL) by heating, then metal magnesium and 5 - 6 drops of concentrated HCl were added. The solution when red was indicative of flavonols and orange for flavones.

**Test** **for** **flavonoids (pew’s** **test).** Five millilitres (5 mL) of the aqueous solution of the water extract was mixed with 0.1 g of metallic zinc and 8ml of concentrated sulphuric acid. The mixture was observed for red colour as indicative of flavonols.

**Test for anthraquinones (Borntrager’s reaction for free anthraquinones).** One gram (1 g) of the powdered seed was placed in a dry test tube and 20 mL of chloroform was added. This was heated in steam bath for 5 min. The extract was filtered while hot and allowed to cool. To the filtrate was added with an equal volume of 10% ammonia solution. This was shaken and the upper aqueous layer was observed for bright pink colouration as indicative of the presence of Anthraquinones. Control test were done by adding 10 mL of 10 % ammonia solution in 5ml chloroform in a test tube.

**Elemental analysis. **The elemental content was determined using the standard calibration curve method.^10^^,^^11^ Zero point (0.5 g) of air dried sample in an evaporating dish was placed in an oven at 80 ˚C and dried to a constant weight. The sample was placed in a weighing crucible and ashed at 500 ˚C in a hot spot furnance for three hours. The ashed material was prepared for the determination of trace element. A portion of zero point (0.5 g) of the ashed sample was digested by heating for two min with a mixture of 10 mL each of nitric acid (HNO_3_), HCl and a perechloric acid in a 500 mL flask. The aliquot obtained from this mixture by filtration was mixed with a 10 mL of 2M HNO_3_ and 30 mL of distilled water in a 100 mL volumetric flask. The volume was made up to zero mark with distilled water. Blank sample and standard solution for the various elements were similarly done. All samples placed in a plastic container and stored in a refrigerator maintained at 4 ˚C prior to analysis. Flame emission spectrometer (Model FGA-330L; Gallenkamp, Weiss, UK) was used to determine sodium (Na) and potassium (K) concentrations. Other elements, magnesium (Mg), calcium (Ca), iron (Fe), lead (Pb), zinc (Zn), manganese (Mn), cadmium (Cd), copper (Cu) and arsenic (As) were determined by atomic absorption spectrometry with (Model SPG No. 1; Unicam, Cambridge, UK) at the appropriate wave-length, temperature and lamp current for each element.^12^

## Results

The phytochemical investigation of the aqueous extract and various solvent fractions revealed that carbohydrate, tannins, saponins, glycosides and flavonoids were present in the extract (Table 1). Anthraquinones, alkaloids, terpene and steroids were absent from the extracts. 

**Table 1:** Phytochemistry of the aqueous, ethyl-acetate, n - butanol and residue fractions of *Acacia nilotica* pods, (+ present; - absent).

**Phytochemical constituent**	**Types of test**	**Aqueous**	**Ethyl-acetate**	**N-Butanol**	**Residue**
**Carbohydrate**	Molisch^’^s Barfoed^’^s Free reducing sugar Combined reducing sugar Ketones Pentoses	+ - + - + +	+ - + - - +	+ - + - - -	+ - + - - -
**Tannins**	Ferric chloride Formaldehyde Chlorogenic	+ + -	+ + -	- + +	+ + -
**Saponins**	Frothing	+	+	+	-
**Glycoside**	General test	+	+	+	-
**Flavonoids**	Lead acetate Sodium hydroxide Ferric chloride Pew	+ - + +	+ - + -	+ - + +	- - - -
**Anthraquinones**	Free anthraquinones Combined anthraquinones	--	--	--	--
**Terpenes and Steroids**	Lieberman – Buchard^’^sSalkwoski’s	--	--	--	--
**Alkaloids**	Dragendorff^’^s Mayer^’^s	--	--	--	--

The concentrations of Fe and K in the *A. nilotica* pod extract were within safety limit. However, the concentrations of some other elements such as Mn, Zn, Ca, Na, Cd and Cu were much lower than the acceptable levels. Elements like Pb, As and Mo were absent (Table 2). The phosphorous and magnesium concentrations in the pod were 0.39 and 0.29 ppm, respectively.

**Table 2:** Elemental analysis of aqueous *Acacia* *nilotica* pod extract

**Elements**	**Concentration ** **(mg dL** ^-1^ ** or ppm)**	**Standard concentration (mg dL** ^-1^ ** or ppm)**
Iron	0.54	0.50 - 50
Potassium	0.52	0.10 - 1.00
Manganese	0.50	10 - 20
Zinc	0.48	15 - 20
Calcium	0.40	360 - 800
Phosphorous	0.39	-
Magnesium	0.29	-
Sodium	0.23	4.00 - 5.00
Cadmium	0.06	10 - 35
Copper	0.04	1.00 - 3.00
Lead	-	1.00 - 2.00
Arsenic	-	0.02 - 7.00
Molybdenum	-	-
Iron	0.54	0.50 - 50

## Discussion

Phytochemical analysis of the aqueous, ethyl acetate and N-butanol fractionated portions of the pod extracts of *A. nilotica *revealed the presence of tannins, saponins, flavonoids, carbohydrate, whereas carbohydrates and tannins were the only constituent in the residue portion. Anthraquinones, alkaloids, terpene and steroids were not present in the extracts (Table 1). The chemical constituents present in the extract have been reported to possess many therapeutic values.^13^^,^^14^

Carbohydrates themselves have not been found to have therapeutic effect, but may possibly increase the effectiveness of the therapeutically important ingredients. Hence a better therapeutic effect may be obtained from the combination of active principles in each plant than by single isolated substance.^15^^,^^16^ Carbohydrates is recently employed in producing polysaccharide immunomodulator with therapeutic and vaccine implications.^17^

Tannins can evoke an antidiarrheal effect and these substances may precipitate proteins on the enterocytes reducing peristaltic movement and intestinal secretion.^18^

Saponins are glycosidic in nature and have expectorant and cardiotonic activity.^9^^,^^19^ Saponins have also been reported to have hypoglycemic and anti-diabetic effects which are very useful in the management of diabetes mellitus.^20^^,^^21^ Saponins found in beans interfere with the replication of cell DNA, thereby preventing the multi-plication of cancer cells.^20^ Glycosides are known to have pronounced physiological action with cardiac glycosides being the drug of choice for the treatment of congestive heart failure.^21^ Furthermore, glycosides are known to have laxative, diuretic and antiseptic properties.^22^^,^^23^

Flavonoids have attracted a great deal of attention due to their potential health benefits. Over the past few years, several experimental studies have demonstrated biological and pharmacological properties of many flavonoids especially their antimicrobial activity,^24^ anti-inflammatory,^25^ antioxidant^26^^,^^27^ and anti-tumour effects^28^ which are associated with free radical-scavenging action. Flavonoids have also been reported to possess hypoglycemic and anti-diabetic effect.^29^ Flavonoids have antioxidant activity, protect cells against oxidative damage and reduce the risk of developing certain types of cancers.^30^

Mineral elements not only serve as nutritional sources for both plant and animals but also play other important roles in the environment. Inorganic chemical elements have been shown to be essential in nutrition and are important structural components in cellular processes.^30^

The concentration of the trace elements like Pb, Zn, Cd, Cu, As, Ca and Mo were either below the acceptable range or absent in the pod extract. Iron was the only trace element found within acceptable level. The study revealed that from all the macronutrients analyzed K was the only one within acceptable range, others like Na and P were below the accepted concentrations.^31^ The heavy elements and essential trace elements are known to influence various body functions based on their concentrations. Elements such as K, Na, Ca, Mg, Mn and Fe play important roles in human and animal health and diseases have been highlighted.^31^

Reportedly, histopathological study of the aqueous pod extract of *A. nilotica* in Wistar albino rats revealed marked hemorrhages in the lungs, heart, kidneys, hepato-cellular necrosis, mononuclear cells infiltration, mild congestion in the stomach and sub mucosal layer of the small intestine. It was therefore concluded that *A. nilotica *pod extract has the potential to produce some level of toxicities when administered at high doses in medicine and veterinary medicine practice.^31^

In conclusion, the phytochemical study revealed the presence of tannins, saponins, glycosides and flavonoids which are compounds capable of causing varied physiochemical and pharmacological effects. Their presence therefore seems to support the traditional use of the plant in the management of various diseases. Heavy trace elements were either absent or below of World Health Organization acceptable levels.
